# Thrombolytic treatment of prosthetic valve thrombosis: a study using Urokinase

**DOI:** 10.1186/s13019-020-01324-7

**Published:** 2020-10-01

**Authors:** Feng Huang, Yongrong Lan, Zhangbo Cheng, Zili Zhang, Fei Ren

**Affiliations:** 1 Department of Cardiovascular Surgery, Fujian Provincial Hospital, Fuzhou, 350000 China; 2 Shengli Clinical Medical College of Fujian Medical University, Fuzhou, 350001 China

**Keywords:** Fibrinolysis, Thrombolytic treatment, Prosthetic valve thrombosis

## Abstract

**Objective:**

We analysed the efficacy and safety of thrombolytic therapy with urokinase in patients with prosthetic valve thrombosis.

**Methods:**

Twenty-three patients with valve thrombosis received thrombolytic treatment using urokinase. First, a 250,000 IU intravenous bolus injection was administered as a loading dose, followed by intravenous infusion of 100,000 IU/h for 10 h and anticoagulation with low molecular weight heparin every day. The maximum treatment time was 5 days, i.e., until the transvalvular pressure gradient was normal or close to normal. Transthoracic echocardiography (TTE) was used every 12 h to monitor whether the thrombus was reduced and whether there was haemodynamic improvement. Routine blood tests, the prothrombin time (PT), international normalized ratio (INR) and complications were observed every day.

**Results:**

Sixteen (69.6%) patients were successfully treated with thrombolytic therapy: 2/2 (100%) aortic valves and 14/21 (66.7%) mitral valves. The partial success rate of this study was 13.0% (3/23). Four patients did not show any improvement in haemodynamics. Two cases had slight urine haemorrhage. One patient died of severe cerebral haemorrhage and shock. The overall mortality was 13.0% (3/23), including two patients who died after subsequent surgery.

**Conclusion:**

Urokinase is more convenient and successful in the treatment of PVT. More experience may make TT the optimal treatment for PVT, especially in high-risk surgical situations.

## Introduction

Prosthetic valve thrombosis (PVT) is a serious complication that occurs after prosthetic heart valve replacement in patients who are poorly anticoagulated. The incidence of thromboembolic complications after mechanical valve replacement is 0.5–8%, especially in patients with poor compliance with anticoagulant therapy [1]. At present, the primary treatment for PVT includes intensive anticoagulation therapy, thrombolytic treatment (TT) and emergency surgery. Compared with that of TT, the mortality rate of emergency surgery in PVT patients is higher. Intravenous TT has been used as an alternative treatment to surgery in PVT patients [2]. We report a single-centre retrospective study of urokinase in patients with PVT to better determine the efficacy and safety of this treatment (Table 1).

**Table 1:** Summary of 23 Cases of PVT

NO	Age(y) Sex	Valve Type	Time from Surgery(m)	NYHA Class	Symptom	UK Dose (0000 IU)	Efficacy	Compli-cations
1	46/M	Mitra/ATS	27	III	Dyspnea	225	CS	No
2	36/M	Mitra /ATS	38	II	Dyspnea	225	CS	No
3	55/F	Mitra /ATS	41	III	APE	325	CS	No
4	49/F	Mitra/CM	1	IV	Dyspnea	225	CS	No
5	67/M	Mitra/ATS	8	III	APE	225	CS	No
6	40/M	Mitra/SJ	97	III	Dyspnea	525	F	No
7	58/M	Mitra /ATS	52	III	APE	525	PS	No
8	62/F	Mitra /ATS	74	IV	APE	425	CS	No
9	65/M	Mitra /ATS	21	III	APE	325	F	Cerebral hemorrhage
10	46/M	Mitra /SJ	18	II	Dyspnea	425	CS	No
11	54/M	Aortic /ATS	27	III	APE	525	PS	No
12	53/F	Mitra /CM	22	III	APE	525	F	No
13	48/M	Mitra /ATS	37	IV	APE	225	CS	No
14	48/M	Mitra /SJ	45	IV	Dyspnea	525	CS	Urine haemorrhage
15	41/M	Mitra /ATS	34	II	Dyspnea	325	CS	No
16	65/F	Aortic /ATS	60	IV	Dyspnea	225	CS	No
17	51/M	Mitra /CM	55	IV	Dyspnea	225	CS	No
18	62/F	Mitra /SJ	43	III	APE	525	CS	Urine haemorrhage
19	46/M	Mitra /ATS	44	IV	Dyspnea	525	F	No
20	44/M	Mitra /SJ	57	III	Dyspnea	325	CS	No
21	39/M	Mitra /CM	16	IV	Dyspnea	325	CS	No
22	58/F	Mitra /ATS	23	III	Dyspnea	525	PS	No
23	45/M	Mitra /SJ	3	III	Dyspnea	225	CS	No

## Methods

Between September 2010 and March 2018, we recruited twenty-three patients treated with fibrinolytic agents whose surgical mortality was considered to be higher than the risk of catastrophic emboli. All patients were confirmed by clinical symptoms, blood examination, electrocardiogram, transoesophageal echocardiography (TEE) and X-ray fluoroscopy. In all cases, fibrinolytic therapy was selected due to no contraindications for fibrinolytic drugs. Contraindications for thrombolysis include the following: 1. Uncontrolled hypertension with blood pressure above 180/110 mmHg; 2. Ischaemic stroke or cerebral haemorrhage within half a year; 3. Intracranial tumour; 4. Active internal bleeding in the last 4 weeks; 5. Suspected aortic dissection; 6. History of major surgical operations in the last 3 weeks; 7. Large vessel puncture at an uncompressible site was performed within 2 weeks; 8. Pregnancy; 9. Active stomach ulcers; and 10. Allergy to thrombolytic drugs. Patients with a thrombus size > 1 cm^2^ or a high density of thrombi were also excluded. There were seven females and sixteen males in this study. The average age of patients with thrombosis was 51.2 ± 9.1 years (36–67 years). The average time between heart valve replacement and thrombus attack was 36.7 ± 23.0 months (1–97 months). There were twenty-one cases of mitral valve prosthesis thrombosis and two cases of aortic valve prosthesis thrombosis. All the involved valves were bileaflet tilting discs (6 Saint Jude, 13 Automatic Transfer Switch, 4 Carbomedics). No patients received adequate anticoagulant therapy when they were diagnosed with PVT.

**Fig. 1:**
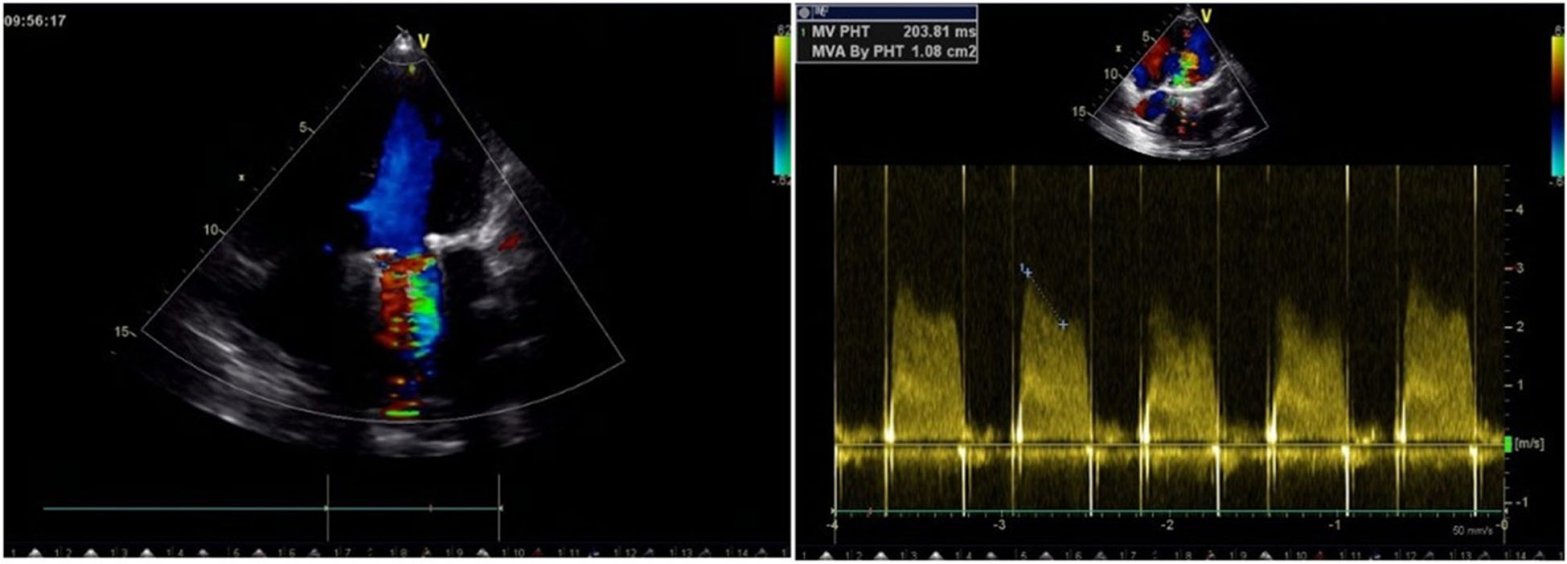
Pre-thrombolysis (mitral valve TEE): effective orifice area (EOA) = 1.08 cm^2^

**Fig. 2:**
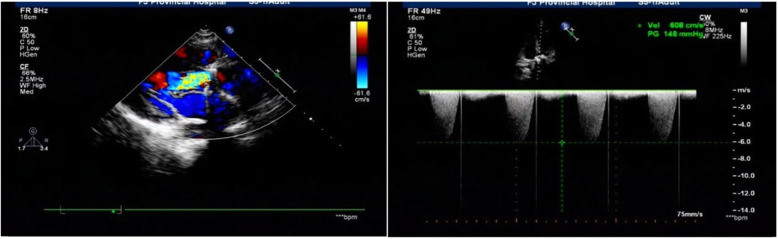
Pre-thrombolysis (Aortic valve TEE): The maximal across valve gradient was 148 mmHg

The main clinical symptoms of PVT are thromboembolism, dyspnoea, and limited activity. Eight patients with acute pulmonary edema (APE) caused by large area occlusion of the prosthesis were classified as New York Heart Association class IV (NYHA IV). Twelve patients were classified as NYHA III, and three patients were classified as NYHA II. Additional investigation of all patients confirmed the clinical suspicion of PVT. TEE was used to diagnose the dysfunction of artificial heart valves, as it can judge the thrombus texture, location, activity and size (Figs. 1 and 2).

### Thrombolytic therapy

All cases were treated with fibrinolysis, regardless of the operation team. First, 250,000 IU intravenous bolus injection was administered as a loading dose, followed by intravenous infusion of 100,000 IU/h for 10 h and anticoagulation with low molecular weight heparin every day. The maximum treatment time was 5 days, i.e., until the transvalvular pressure gradient was normal or close to normal. Transthoracic echocardiography (TTE) was used every 12 h to monitor whether the thrombus was reduced and whether there was haemodynamic improvement. Routine blood tests, the prothrombin time (PT), international normalized ratio (INR) and complications were observed every day.

Complete success was considered to have normal or near-normal cross-valve gradients restored without any serious complications. Partial success means that the cross-valve gradient was reduced by more than 50% or the haemodynamics were significantly improved without any serious complications. The failure of TT was considered to be no significant improvement in valve activity and the cross-valve gradient after treatment or serious complications during thrombolysis.

## Results

### Efficacy of Fibrinolytic treatment

**Fig. 3:**
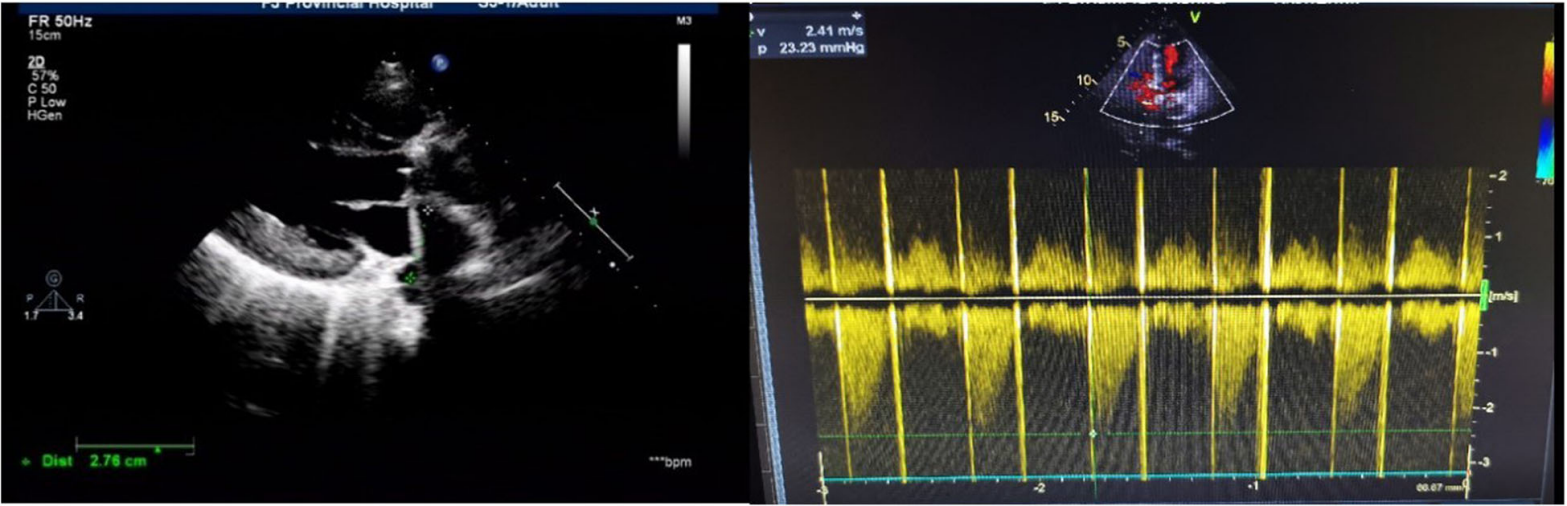
Post-thrombolysis (Aortic valve TTE): The maximal across valve gradient was 23.23 mmHg

**Fig. 4:**
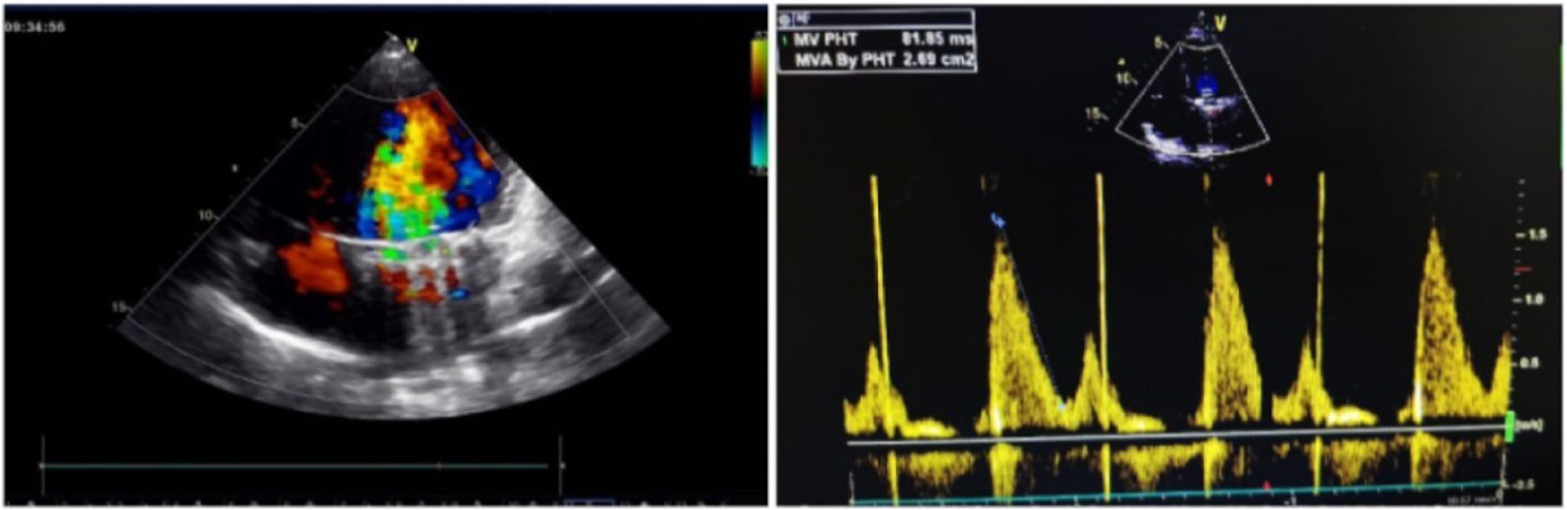
Post-thrombolysis (mitral valve TTE): EOA = 2.69 cm^2^

Of the twenty-three patients, sixteen (69.6%) were successfully treated with thrombolytic therapy: 2/2 (100%) aortic valves (Fig. 3) and 14/21 (66.7%) mitral valves (Fig. 4). The partial success rate of this study was 13.0% (3/23), and these patients received 5 cycles of UK treatment. However, even after 5 cycles, four patients did not show any improvement in haemodynamics. Except for one patient who died of cerebral haemorrhage, the other six patients underwent thrombolysis due to incomplete success or failure of fibrinolytic treatment.

### Dosage of Urokinase

The dosage of urokinase in patients with successful thrombolysis ranged from 2,250,000 IU to 5,250,000 IU. However, most patients with successful thrombolysis used 2,250,000 IU or 3,250,000 IU of urokinase.

The dosage of urokinase in other patients was 5,250,000 IU.

### Complications of Fibrinolytic therapy

Haemorrhagic complications: Two patients had slight haematuria. One patient died of severe cerebral haemorrhage and shock.

Embolic episodes: No thromboembolic complications occurred in any of the patients.

The overall mortality was 13.0% (3/23), including two patients who died after subsequent surgery. There was no obvious relationship between cardiac function classification and complications. However, all patients who died were classified as NYHA functional grade IV.

## Discussion

The treatment for PVT includes intensive anticoagulation therapy, TT and emergency surgery. The effectiveness of PVT anticoagulant therapy has been examined in only a few publications [3–5]. It has been reported that the treatment of small asymptomatic thrombi (length < 10 mm) by optimizing anticoagulant therapy has a good prognosis [6]. These patients had a high mortality rate during TT and surgery. In the past few decades, thrombolytic therapy has been increasingly used in PVT. Although there have been numerous reports worldwide on the clinical manifestations in and treatment options and methods for patients with artificial valve thrombosis, the best treatment is still controversial. Treatment options depend on many factors, such as the presence of valve obstruction, the patient’s clinical condition, the size of the thrombus, the local medical and economic level, and the experience with reoperation. Karthikeyan analysed 690 cases in seven PVT thrombolysis studies and found no significant difference in major outcomes (improvement in transvalvular pressure gradient and serious complications) between surgery and thrombolysis [7]. However, they suggested that an emergency surgical intervention in an experienced centre is preferable to TT. Compared to this meta-analysis, previous data showed that the mortality rate for surgery was as high as 69%, while the reported mortality rate for TT was as high as 16%, depending on the NYHA grade and the urgency of the surgery [8]. Therefore, surgical treatment is not suitable for all patients. Limited availability and high surgical costs have made TT the optimal treatment for PVT in hospitals that do not have reoperation experience or are located in economically underdeveloped areas.

Several fibrinolytic medicines commonly used in previous studies, such as urokinase, streptokinase, tenecteplase and recombinant plasminogen activator, have different therapeutic effects and different levels of safety in PVT. Recombinant plasminogen activator and tenecteplase thrombolytic therapy for PVT has been widely used in many studies, but no one has reported it in China. Urokinase is still commonly used in clinical practice in China due to its low cost and convenience. Several studies have shown that urokinase is less effective in the treatment of PVT than streptokinase or recombinant plasminogen activator [9–11]. Raymond Roudaut reported that only 36.6% of patients treated with urokinase (UK group) were successfully treated [12]. In this study, urokinase was used to treat PVT, with a complete success rate of 69.6% and a partial success rate of 13.0%. The success rate of thrombolysis in this study was significantly higher than that reported previously. In this study, all patients had poor drug compliance and were examined by transoesophageal ultrasound before TT, which reduced the possibility of non-thrombotic valve obstruction and improved the success rate of thrombolysis. This may also be related to the fact that the duration of urokinase use (up to 5 days) and dose (up to 5.25 million IU) were both higher in this study than in previous studies.

The major complications of thrombolytic therapy are haemorrhage and embolism. The incidence of complications is different due to the various drugs and methods used for thrombolysis. Most bleeding complications are usually benign. In the absence of potential bleeding tendencies, such as pregnancy, postoperative status, or pericarditis, bleeding during fibrinolysis is rare [13, 14]. In this study, there were three cases of haemorrhage (13.0%), including one case of severe cerebral haemorrhage (4.35%) and two cases of urinary system haemorrhage. Lengyel reported a 5% incidence of major bleeding, which is less than that in our study but consistent with another study [15].

Cáceres-LórigaI reported a 4.6 to 12.8% incidence of embolism events in a meta-analysis, which included nine studies with 413 patients [16]. No embolic events were observed in this study. An ultralow rate infusion strategy can gradually dissolve a thrombus and avoid the occurrence of embolism caused by rapid thrombolysis of the thrombus. Although high-speed infusion therapy may accelerate thrombolysis and restore haemodynamics more quickly, it also increases the risk of severe thromboembolism and bleeding events. Recently, the 2017 AHA/ACC Focused Update of the 2014 AHA/ACC Guideline for the Management of Patients with Valvular Heart Disease recommended using slow-infusion low-dose TT or emergency surgery for obstructive PVT as the first-line treatment strategy for class 1-B indications [17].

TEE is crucial in diagnosing artificial valve dysfunction, especially in the diagnosis and treatment of PVT. When TTE or CF cannot make an accurate diagnosis, TEE examination can be used to confirm the diagnosis and make the best treatment plan. Therefore, TEE has been used to diagnose whether artificial valve thrombosis causes obstruction and is particularly important in assessing thrombus size, mobility, and location [18, 19]. Large thrombi can lead to thromboembolic events, heart failure, and even cardiac arrest. Therefore, the thrombus size measured by TEE is crucial in determining the best treatment strategy. In this study, most of the prosthetic valve thromboses were less than 1.0 cm^2^, so the success rate of thrombolysis was higher than it was previously.

## Conclusion

Urokinase is more convenient and successful in the treatment of PVT. More experience may make TT the optimal treatment for PVT, especially in high-risk surgical situations. Furthermore, the advent of new thrombolytic drugs and new diagnostic methods make thrombolytic therapy for PVT more effective and safer.

## Data Availability

Not applicable.

## Abbreviations

PVT: Prosthetic valve thrombosis
TT: Thrombolytic treatment
PT: Prothrombin time
INR: International normalized ratio
F: Female
M: Male
SJ: St. Jude
CM: Carbomedics
UK: Urokinase
APE: Acute pulmonary embolism
CS: Complete success
PS: Partial success
F: Failure
TEE: Transesophageal echocardiography
NYHA: New York Heart Association class
TTE: Transthoracic echocardiography
